# An engineered CARS substrate with giant field enhancement in crisscross dimer nanostructure

**DOI:** 10.1038/s41598-017-18821-w

**Published:** 2018-01-15

**Authors:** Jia Zhang, Shu Chen, Junqiao Wang, Kaijun Mu, Chunzhen Fan, Erjun Liang, Pei Ding

**Affiliations:** 1 School of Physical Science and Engineering and Key Laboratory of Materials Physics of Ministry of Education of China, Zhengzhou University, Zhengzhou, 450001 China; 2 Department of Mathematics and Physics, Zhengzhou Institute of Aeronautical Industry Management, Zhengzhou, 450015 China

**Keywords:** Metamaterials, Nanophotonics and plasmonics

## Abstract

We theoretically investigate the optical properties of a nanostructure consisting of the two identical and symmetrically arranged crisscrosses. A plasmonic Fano resonance is induced by a strong interplay between bright mode and dark modes, where the bright mode is due to electric dipole resonance while dark modes originate from the magnetic dipole induced by LC resonances. In this article, we find that the electric field “hotspots” corresponding to three different wavelengths can be positioned at the same spatial position, and its spectral tunability is achieved by changing geometric parameters. The crisscrosses system can be designed as a plasmonic substrate for enhancing Coherent Anti-Stokes Raman Scattering (CARS) signal. This discovery provides a new method to achieve single molecule detection. At the same time, it also has many important applications for multi-photon imaging and other nonlinear optical processes, such as four-wave mixing and stimulated Raman scattering.

## Introduction

A common coupling characteristic for metallic plasmonic nanostructures is the Fano-type resonance with a distinct asymmetric line shape. The plasmonic Fano resonance arises from the coherent interference between the narrowband dark plasmonic mode and the broadband bright plasmonic mode^1^, which has attracted broad research interests due to its significant applications such as electromagnetically induced transparency (EIT)^2,3^, optical switching^4,5^, surface enhanced Raman scattering (SERS)^6,7^, biological sensors^8,9^, nonlinear^10–12^ and slow-light optical device^13^. In the complex metal nanostructures, plasmonic interferences and hybridizations provide a strong strategy to intensify local field enhancements and tailor the spectral response at nanometer scale.

So far, many studies have been focused on plasmonic nanostructures that exhibit Fano resonance depending on various excitation ways. In coupled metamaterial structures, structurally configurations take astonishing roles for modifying the electromagnetic property. The most common way is introducing asymmetric geometry structure. It is the planar ring-disk or three-dimensional sphere-shell metallic nanostructures that can exhibit a sharp Fano resonance by introducing a coupling between dipolar modes (bright) and the quadrupolar modes (dark) through shifting the center particle with respect to the center of the surrounding ring or shell^14,15^. In nanoparticle pairs, the coupling will be introduced to produce Fano resonances, when we change the size or composition of the one of nanoparticles. For instance, the asymmetric nanorod pairs structure^16^ with a length offset *l* can exhibit a Fano resonance due to the strong interference between the electric dipole resonance and the magnetic dipole resonance. Another type of symmetry breaking is the introduction of asymmetric dielectric environment or substrate^17,18^. The Fano resonance has been experimentally observed in gold nanorods structure supported on a substrate with very high dielectric constant^19^. Furthermore, the Fano resonance in metal nanostructures can also be obtained in a variety of arrays^20–22^ or grating structures^23^. This is mainly due to the interaction between narrow diffraction resonance and wide plasmonic excitation modes. Metal nanostructure is one of the most important elements to construct the plasmonic compounds, and plays an important role in promoting the rapid growth of the plasmonic metamaterials.

Recently, metallic plasmonic nanostructures are of particular interest for the detection and identification of single molecules using Fano resonance. Halas research group^24^ had proposed that the coherent anti-Stokes Raman spectroscopy (CARS) can be applied to acquire single molecule detection sensitivity based on a Fano substrate. This model explains in detail how it observes the four-wave mixing, which provides a new strategy for implementing the third-order process in nanostructures. Subsequently, He^25^ present a design consisted of three asymmetric gold disks with large local field enhancement. It is interesting to achieve a high enhancement factor about ~10^13^ as a CARS substrate.

Recently, we have theoretically investigated the plasmonic film-crisscross dimer array system^26^, which can be acted as a multi-resonance plasmonic substrate. In this structure, the three plasmonic resonance modes excited in the visible or near infrared spectral range can be matched with the pump light, Stokes and anti-Stokes lights, respectively, to achieve a significant amplification of the CARS signal. The mainly factors to achieve this purpose are that this film-crisscross dimer substrate can excite multiple plasmonic resonances with strong local characteristics, all of them have same hotspot positions, and the spectral will overlap between plasmonic resonances and input beams or output beams. It is the multi-resonance plasmonic substrate that will be more conducive to the realization of enhanced nonlinear spectroscopy and imaging.

In this paper, we continue to investigate in-depth this crisscross-dimer structure consisted of two identical and symmetrically arranged crisscrosses in a scattering system. Here, we mainly research the scattering performance of crisscross dimer by changing the structural parameters to flexibly adjust the resonance spectra, and using circuit model theory to analyze and predict the resonance characteristics. Scattering spectra with Fano-type profile is excited in the crisscrosses structure. Furthermore, another resonance peak will appear, when the arm length of *l*_2_ and *l*_3_ is unequal. This find provides a new tactic for the design of multi-resonances plasmonic CARS substrate, and the surface enhanced CARS signal can reach about ~10^13^. Although the CARS enhancement in the scattering system is less than that our study of film-crisscross dimer array structure, it can still reach the level of previous research by other scholars, which still has an important guiding significance for single molecule detection.

## Structures Description

**Figure 1:**
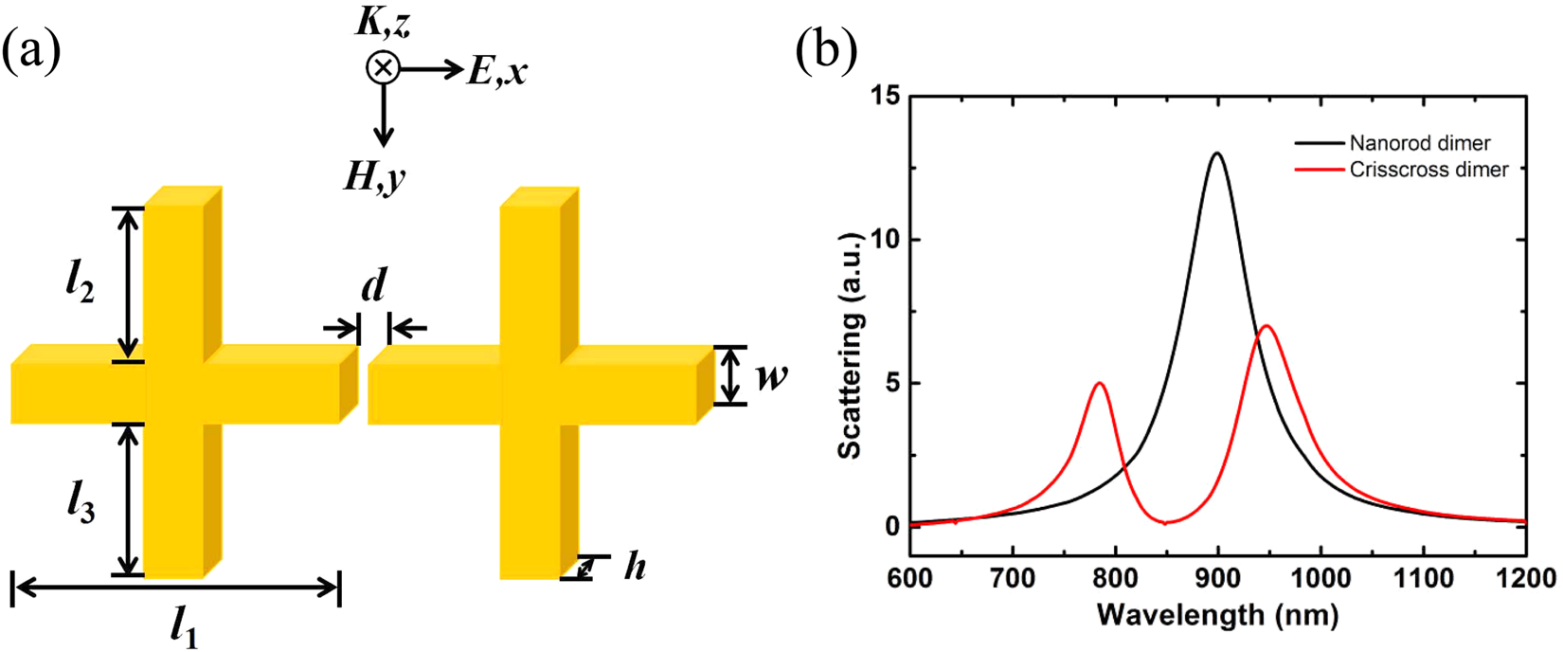
(**a**) Sketch of the designed two identical crisscrosses structure with a incident plane wave at the *z* axis direction and the electric fields polarized along the *x* direction; (**b**) Scattering spectra for nanorod dimer (black) and crisscross dimer (red).

In this study, we use crisscross dimer as a metamaterial design platform to study scattering effects, coupling strength and symmetry breaking. The schematic drawing of the crisscross dimer is shown in Fig. 1(a), consisting of two identical crisscrosses arranged symmetrically about the *y*-axis, with the subunit length of *l*_1_, *l*_2_ and *l*_3_, where *l*_1_ = 120 nm, the width *w* = 30 nm, the thickness *h* = 20 nm, the spacing gap between two crisscrosses *d* = 10 nm is kept constant, and the length of *l*_2_ and *l*_3_ can be changed. In the simulation process, we select the parameter m = *l*_2_ − *l*_3_ as a variable to describe the asymmetric system. A plane electromagnetic wave is incident along the *z*-direction with the electric field polarized along the *x*-direction. The scattering property and field distribution in proposed structure are realized by finite element analysis method with the physical simulation function of COMSOL multiphysics software. The solution domain is composed of perfect match layers (PMLs) as boundary condition for all six boundaries. Silver is chosen as crisscross dimer nanostructure, surrounded by vacuum.

## Results and Discussions

### Crisscrosses structure

For the crisscross dimer structure in scattering system, we mainly study its scattering properties by adjusting the length of *l*_2_ and *l*_3_. When *l*_2_ = *l*_3_ = 0, there exist only a nanorod pair with a gap *d* and arm length *l*_1_, which is also known as the end-end nanorod dimer^27^, which is effectively excited by the incident light to provide a bonding dipole resonance. Its scattering spectra exhibits a standard Lorenz lineshape, as shown in Fig. 1(b) for the black curve. However, when adjusting *l*_2_ = *l*_3_ ≠ 0, an asymmetric Fano resonance occurs due to the destructive interference between bonding dipole resonance and the magnetic resonance, which is shown in Fig. 1(b) for the red curve.

**Figure 2:**
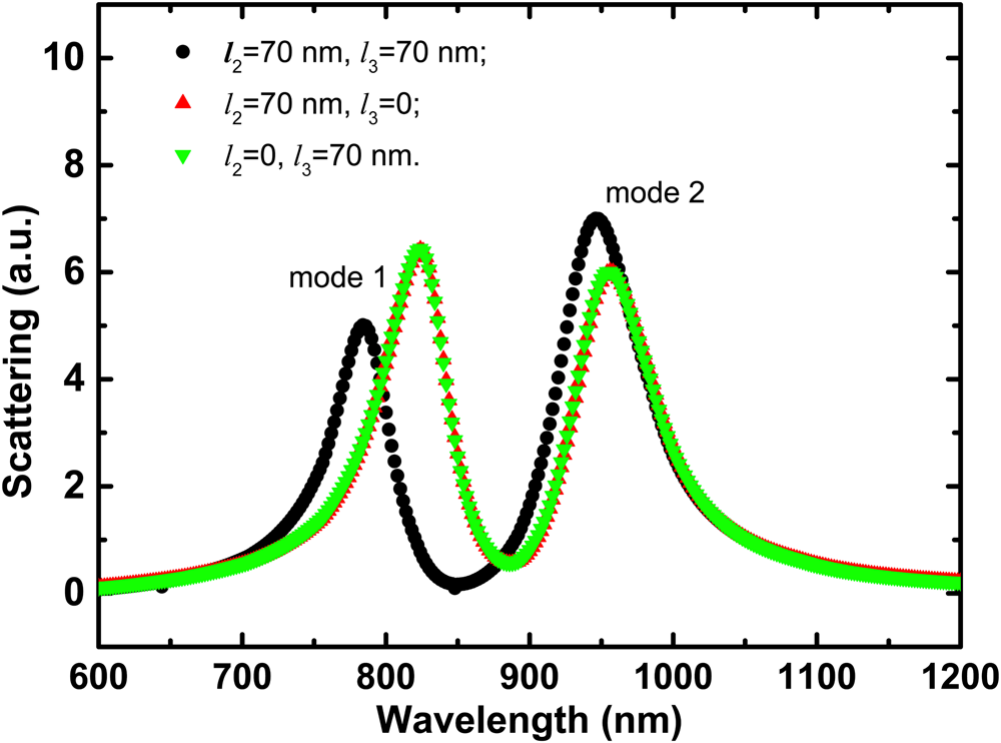
Scattering spectra for the structures of *l*_2_ = *l*_3_ = 70 nm (black); *l*_2_ = 70 nm, *l*_3_ = 0 (red); and *l*_2_ = 0, *l*_3_ = 70 nm (green).

**Figure 3:**
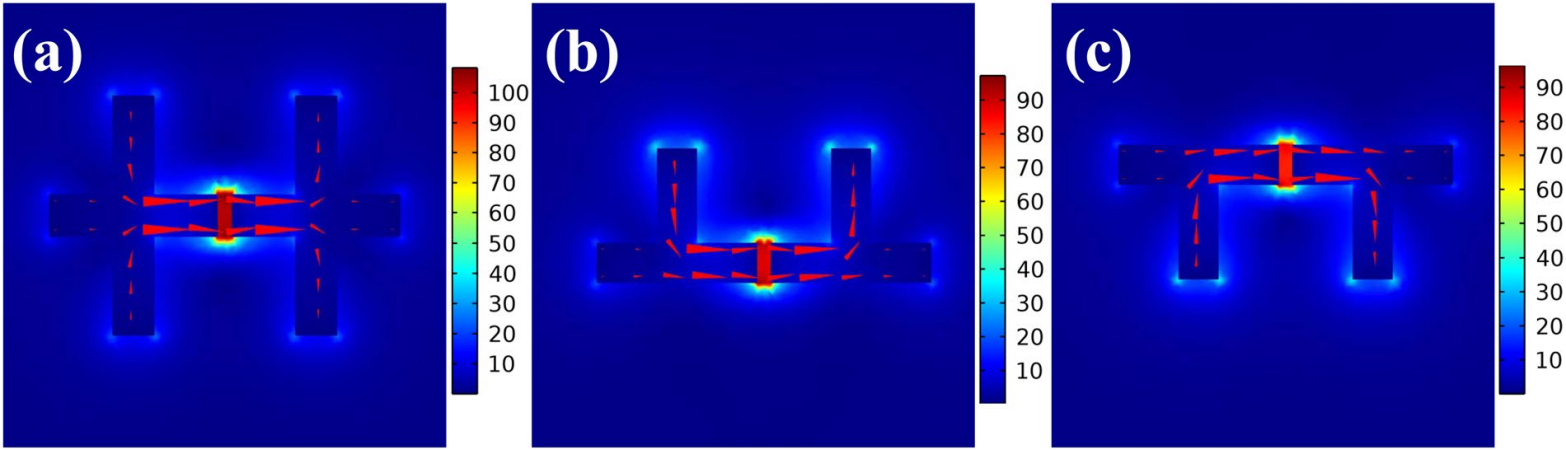
Simulated electric field and current flow distribution of the crisscrosses at *mode 2* with different values of *l*_2_ and *l*_3_: (**a**) *l*_2_ = *l*_3_ = 70 nm; (**b**) *l*_2_ = 70 nm, *l*_3_ = 0; (**c**) *l*_2_ = 0, *l*_3_ = 70 nm. The red arrows represent the induced current flow direction and the color bars represent the electric field density.

In order to well analyze the optical response for the crisscross dimer structure, we first research the crisscross dimer with *l*_2_ = *l*_3_ = 70 nm, while the other parameters are the same as the initial settings. The black curve in Fig. 2 shows measured normal incidence, horizontal polarization scattering spectra. The short wavelength peak marked with *mode 1* is derived from the bonding dipole resonant depending on the antenna pairs of arm length *l*_1_. The other peak marked with *mode 2* is typically produced to a LC resonant behavior, due to an antibonding magnetic dipole resonance. In this article, we will mainly analyze this magnetic dipole resonance *mode 2*. As shown in Fig. 3(a), the electric field is concentrated in the gap between two crisscrosses. There are two current loops are located at the bottom and the top of the crisscrosses, depending on the anti-parallel current distribution in the crisscrosses. This resonance is therefore attributed to the out-of-phase magnetic dipole oscillations. It is analogous to LC resonance in SRR or U structure^28,29^. Nanorods serve as inductance L and the gap *d* between the crisscross dimer provide a capacitance C.

### Equivalent circuit analysis of crisscrosses structure

In our designed nanostructures, the two crisscrosses are identical and symmetrically placed with respect to *y-*axis. In LC resonant *mode 2*, the structural elements in parallel with the *y*-axis play an important role. For the crisscross dimer, as shown in Fig. 3(a), the current direction in the subunit of the length *l*_2_ is opposite to that of the subunit with the length *l*_3_. Moreover, the current directions of the same part between the two crisscrosses are also reversed. Thus, two current loops are formed in the bottom and the top parts of the crisscross dimer. Based on the above analysis, we assume that the resonance *mode 2* can be determined by the bottom and top parts, respectively.

**Figure 4:**
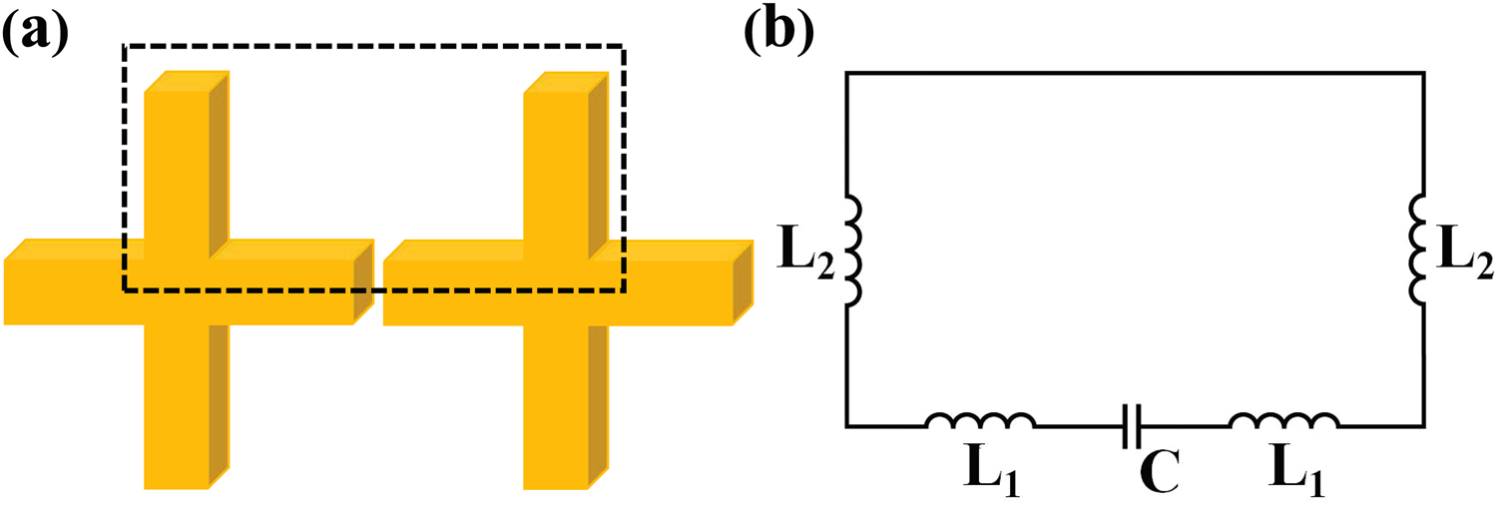
(**a**) The equivalent structure of the crisscrosses can be transformed into the part of the dashed box for LC resonance *mode 2*; (**b**) Simplified LC equivalent circuit model of the crisscross dimer.

To verify our assumption, we also consider the other two cases: (a) *l*_2_ = 70 nm, *l*_3_ = 0 and (b) *l*_2_ = 0, *l*_3_ = 70 nm, while maintaining other parameters unchanged. Their corresponding scattering spectra are shown by the red curve and the green curve in Fig. 2, respectively. In contrast to the crisscross dimer, the resonant *mode 1* has a significant redshift in each of the upper or lower structures, which is primarily affected by the subunit length in the *y* direction^30^. For the resonant *mode 2*, the resonant position does not change substantially, except that the scattering intensity becomes smaller. The Fig. 3(b),(c) illustrate the electric field and current flow distribution in the upper or lower parts of the crisscrosses. They have same resonance frequency and electric field distribution, and correspond to the dimer structure. So we can only consider the part of the dashed box in Fig. 4(a). A simple equivalent circuit model is established as shown in Fig. 4(b).

So far, the inductor-capacitor (LC) circuit model has been applied to predict the plasmonic resonance frequency^31^. It is useful to understand physical mechanisms and the geometric effects in nanostructure metamaterial. The electric field intensity distribution shows a large number of charge accumulations in the gap in *mode 2*. By adding successive image charges method proposed by Corrigan^32^, the coupling capacitance C can be approximated as:

1 $$C\cong 4\pi \varepsilon a(1+\frac{{a}^{2}}{{{\rm{g}}}^{2}-{a}^{2}}+\frac{{a}^{4}}{{g}^{4}-3{g}^{2}{a}^{2}+{a}^{4}})$$

where, the capacitor is formed at the gap, *ε* is the permittivity of free space, *a* is the equivalent radius of the sphere and *g* is the center spacing of the isolated spheres. The equivalent sphere essentially originates from the same volume of the cylinder with a cross-sectional radius *r* and a height *2r*.

The inductance of the current carrying can be calculated by the Bueno theory^33^:

2 $${\rm{\Lambda }}(l)=\frac{{\mu }_{0}l}{4\pi }[2{\sinh }^{-1}(\frac{l}{w})+(1+k)(\frac{l}{w}){\sinh }^{-1}(\frac{w}{l})-\frac{(3-k)}{3}\frac{{({w}^{2}+{l}^{2})}^{3/2}}{l{w}^{2}}+({\rm{1}}-k)\frac{l}{{w}^{2}}{(w{}^{2}+{l}^{2})}^{1/2}+\frac{2k}{3}{(\frac{l}{w})}^{2}+\frac{(3-k)}{3}(\frac{w}{l})]$$

where *l* is the length of nanostructure, µ_0_ is the permeability of free space, and *k* is an integer number, according to the Neumann model, *k* = 1 is selected during the calculation. Thus, the self-inductance in our proposed structure can be simply expressed as: ${L}_{{\rm{1}}}={\rm{\Lambda }}({l}_{1}/2)$, ${L}_{2}={\rm{\Lambda }}({l}_{2}/{\rm{2}})$. In addition, the mutual inductance between the nanorods is expressed as:

3 $$M(l,g)=-\frac{{\mu }_{0}}{4\pi }[2l{\sinh }^{-1}(\frac{l}{g})+(3-k)(g-{({g}^{2}+{l}^{2})}^{1/2})]$$

The negative sign indicates that the direction of the current is reversed, and *g* is the distance between nanorods. To simplify the circuit model based on equivalent structure in Fig. 4, the inductance of the entire loop can be expressed as: $L=2{L}_{1}+2{L}_{2}-2M$. So the resonant wavelength *λ*_*r*_ in a single closed loop can be extracted through the relation:

4 $${\lambda }_{r}=2c\pi \sqrt{LC}$$

### Fano resonance in symmetric crisscrosses structure

**Figure 5:**
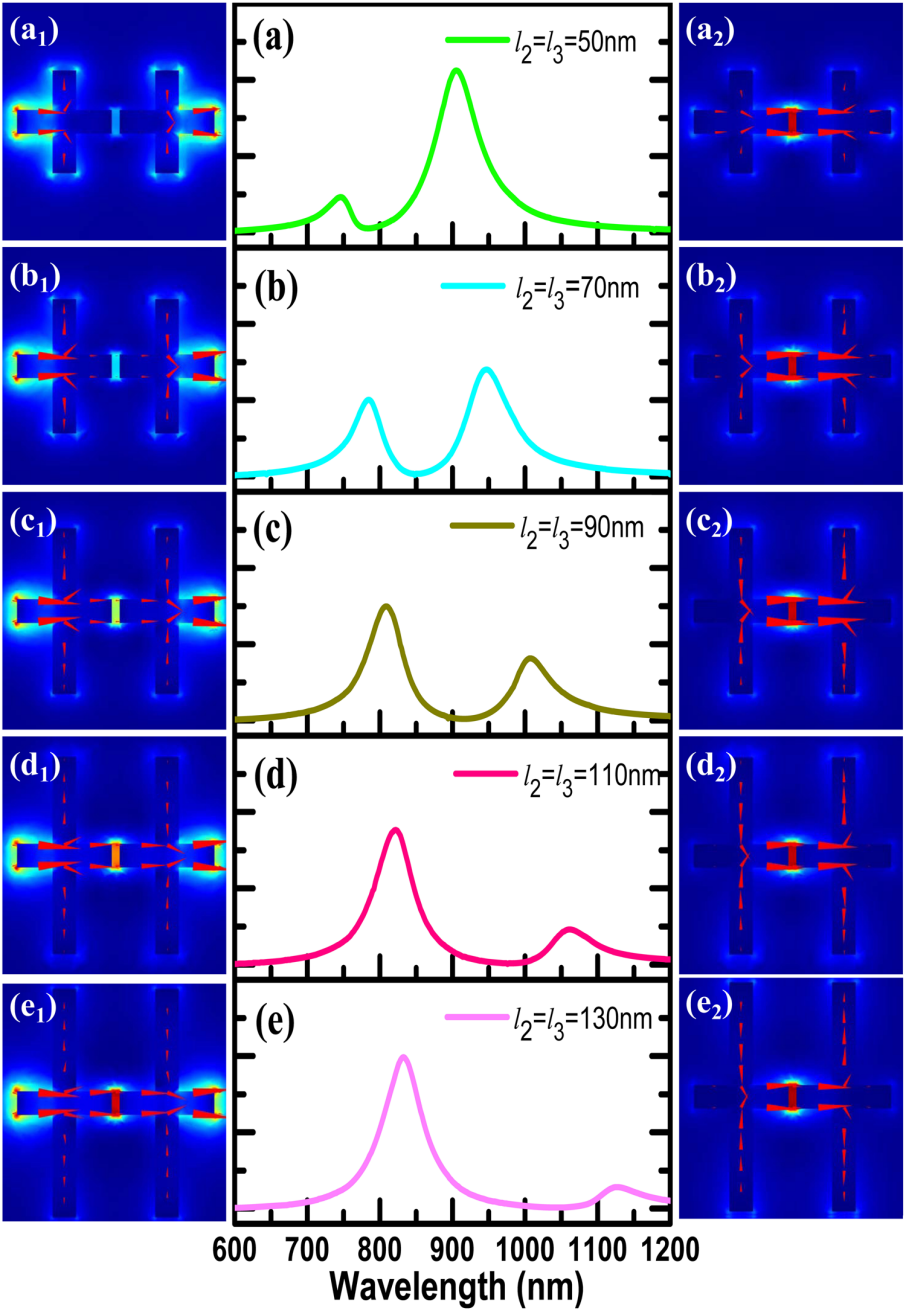
(**a**–**e**) Simulated scattering resonance properties for the *l*_2_ = *l*_3_ = 50, 70, 90, 110, 130 (nm); (a_1_–e_1_) and (a_2_–e_2_) The electric field intensity and current distribution for resonance *mode 1* and *mode 2*, respectively.

**Figure 6:**
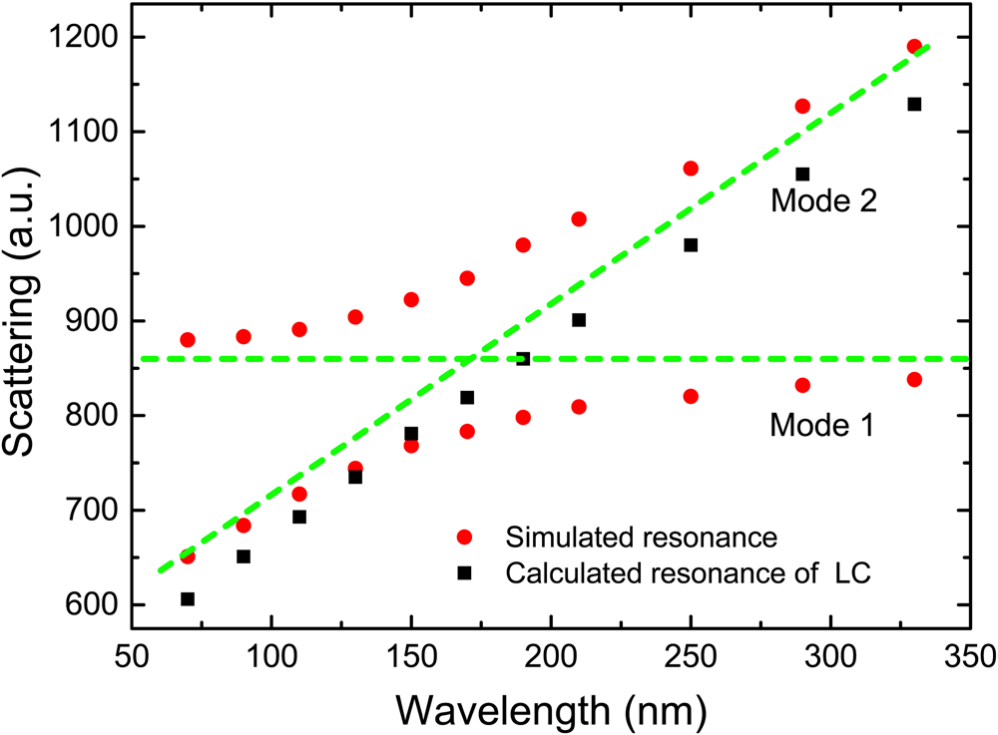
Dependence of resonance wavelength on the length *l*_2_ and *l*_3_. The red dots indicate the simulated resonant *mode 1* and *mode 2*, the black rectangles represent the calculated resonance.

The LC circuit model would be useful to predict how the resonances shift as the varied shape and position of the elements. In order to verify its effectiveness, we have rigorous simulation calculations. We flexibly change the length of *l*_2_ and *l*_3_, so that their values are *l*_2_ = *l*_3_ = 50, 70, 90, 110, 130 (nm), and other parameters remain unchanged. In the symmetrical crisscross dimer, we simply express structural adjustment by describing the changes in *l*_2_ value instead of the description of *l*_2_ and *l*_3_. When the length of *l*_2_ is gradually increased, the resonance scattering spectra is shown in Fig. 5(a–e). The interaction between the electric dipole resonance and the magnetic dipole resonance leads to a significant Fano resonance. Simultaneously, the spectral line shape can be flexibly controlled with the increase of *l*_2_. The resonance wavelength dependence on *l*_2_ is shown in Fig. 6. The red dots indicate the simulated resonant mode, and the black rectangles represent the calculated LC resonance mode. With the length of *l*_2_ gradually increasing, when the magnetic dipole resonant mode induced by the subunits *l*_2_ matches with electric dipole the resonance provided by the nanorod dimmer of length *l*_1_, the two modes will produce strong coupling phenomenon and linewidth of the coupled states appears obvious intersect splitting phenomenon, as shown in the anti-crossing region. The spectral line shape can be flexibly controlled with the adjustment of *l*_2_. However, when the length of *l*_2_ continues to increase, the two modes will be independent of each other, keeping their own characteristics due to their mismatch. According to formula (4), the LC resonant mode we calculated is in good agreement with the simulation, as shown in the black rectangles in Fig. 6. So this method can be well used to predict the resonance *mode 2* by adjusting the length of *l*_2_.

Figure 5 (a_1_–e_1_) and (a_2_–e_2_) depict the electric field intensity and current density distributions of *modes 1* and *mode 2*, respectively. It can be seen from the *modes 1* in Fig. 5(a_1_–e_1_), bonding dipoles are formed in the bottom and top parts in the crisscross dimer. When the *l*_2_ length is relatively short, the electric field “hotspot” is mainly concentrated at the end of the end-end nanorod dimer due to the influence of the subunit *l*_2_, and the current density distribution is mainly around the outer corners (from *l*_1_ to *l*_2_ or vice versa). With the increase of *l*_2_, the electric field “hotspot” moves from the end to the middle gap, obviously. It is important for us to realize the same “hotspot” distribution. Furthermore, for the *mode 2* in Fig. 5(a_2_–e_2_), it originates from the opposite charge flow between two subunits of the length *l*_2_, which builds up intense magnetic fields. It is clear that the electric field “hotspot” is always localized at the gap position.

### Multi-plasmon resonance in asymmetric crisscross dimer

**Figure 7:**
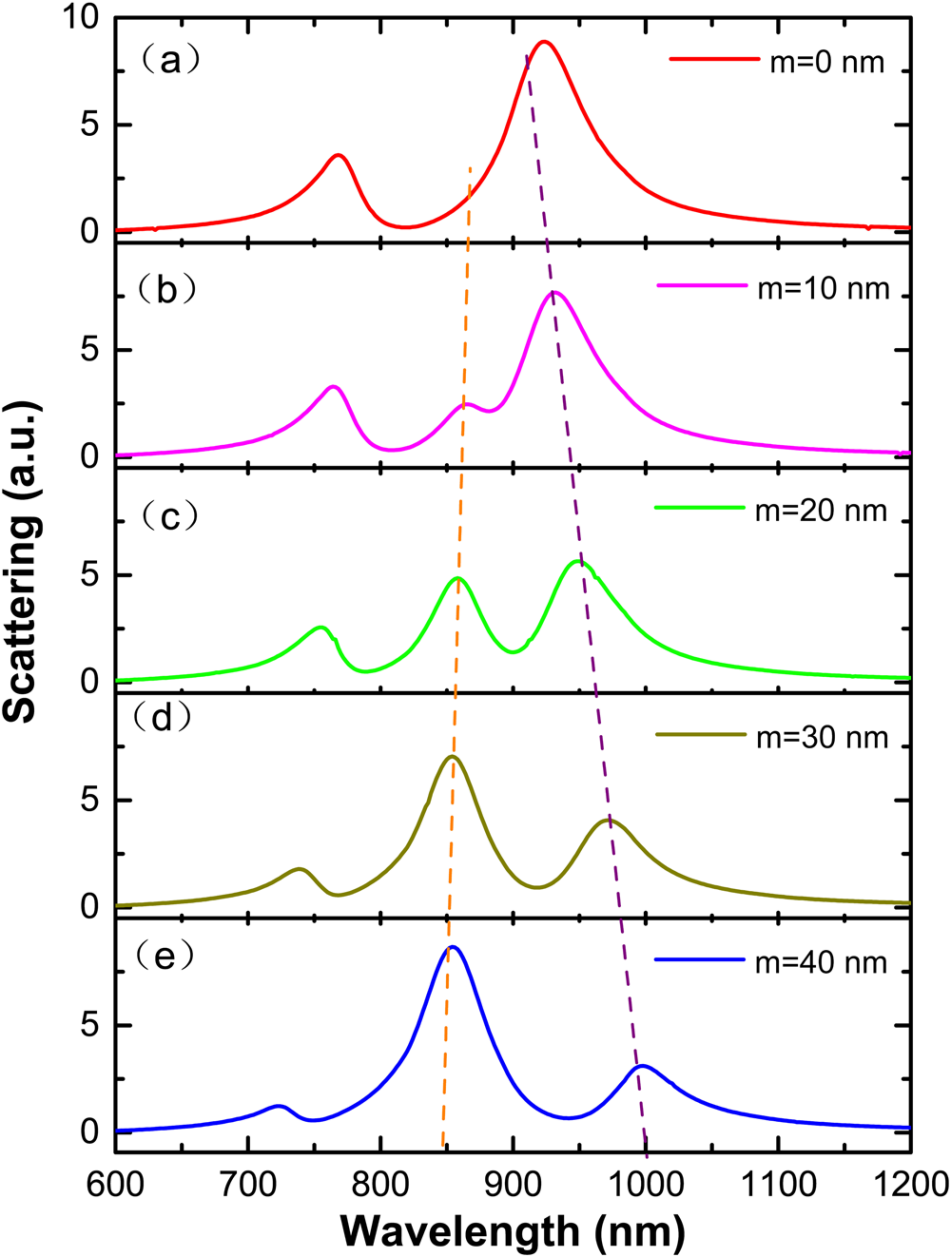
Simulated scattering resonance properties in asymmetry crisscross dimer with different values between *l*_2_ and *l*_3_. The sum of *l*_2_ and *l*_3_ is a fixed value of 150 nm, *m* represents the difference between *l*_2_ and *l*_3_ (*m* = *l*_2_ − *l*_3_).

Obviously, structural symmetry has not been destroyed in our designed crisscross dimer. It has been proved that the symmetric structure can produce a Fano-like resonance. However, another striking spectral characteristic is found in Fig. 7. In the crisscross dimer, when the sum of *l*_2_ and *l*_3_ is a fixed value of 150 nm (shown in Fig. 7(a)), the resonant spectra exhibits a clear asymmetric Fano line shape due to the interaction between the magnetic dipole and the electric dipole, as described above. However, we do not change the sum of *l*_2_ and *l*_3_, only adjust the length of *l*_2_ and *l*_3_ to make the difference *m* = *l*_2_ − *l*_3_. The scattering performance is shown in Fig. 7(b–e). When *m* is not equal to 0, the magnetic dipole moments at the bottom and the top parts of the crisscross dimer are unequal, so that the resonance *mode 2* splits to appear an additional resonance peak^30^. This result indicates that the spectral linetype can be adjusted by changing the *m* value, and it provides a new method for achieving multiple plasmonic resonance.

### The SECARS based on crisscross dimer

Recently, the development of substrate has provided tremendous impetus for surface-enhanced spectroscopy studies. CARS has also been widely studied to enable the detection of single molecules^35^ and the design of surface enhanced spectroscopic substrate^36^. We are known that it is a third-order nonlinear optical process. When the pump beam with frequency ω_p_ and the Stokes beam with frequency *ω*_s_ interact with the medium and the frequency difference *ω*_p_−*ω*_s_ satisfies the Raman dipole-forbidden band distribution condition of the molecule, it will lead to the generation of an anti-Stokes signal with frequency *ω*_as_. The CARS signal will result in resonance enhancement at the position of the resonance peak. The enhanced CARS signal may be realized using a metal plasmonic substrate, which can be regarded as surface enhanced CARS (SECARS). The electromagnetic field enhancement factor (EF) at the anti-Stokes frequency is expressed as:

5 $${G}_{SECARS}={|E({\omega }_{p})/{E}_{0}({\omega }_{p})|}^{4}\times {|E({\omega }_{s})/{E}_{0}({\omega }_{s})|}^{2}\times {|E({\omega }_{as})/{E}_{0}({\omega }_{as})|}^{2}={{g}_{p}}^{4}\times {{g}_{s}}^{2}\times {{g}_{as}}^{2}$$

Which implies that the CARS signal enhancement of local electromagnetic field is determined by three characteristic frequencies. The *g*_*p*_, *g*_*s*_ and *g*_*as*_ are the enhancements of the localized electric fields at the pump($|E({\omega }_{p})/{E}_{0}({\omega }_{p})|$), Stokes($|E({\omega }_{s})/{E}_{0}({\omega }_{s})|$) and anti-Stokes ($|E({\omega }_{as})/{E}_{0}({\omega }_{as})|$) resonance, respectively. Thus a suitable plasmonic substrate that achieves the CARS signal will provide strong electromagnetic field depending on three characteristic frequencies. If the three resonant modes that form the “mixed frequency coherent mode” have same spatial positions. It will be useful as a CARS substrate to achieve a strong field enhancement. Simultaneously, the surface plasmonic resonance plays a crucial role in exciting extremely large intensities near the metal surface and field localization is always induced by constructive coherences in nanostructures^37^.

In our designed the crisscross dimer structure, when the length of *l*_2_ and *l*_3_ is adjusted so that they are not equal, three different plasmonic resonances can be achieved. In order to achieve large CARS signal enhancement, it is necessary to localize the “hotspot” positions of the three resonant frequencies at the same spatial location. Only in this way can the maximum resonance enhancement be realized. Based on CARS performance studies of crisscross dimer nanostructures, we matched and achieved CARS signal enhancement for the different configuration parameters by adjusting the length of nanorods *l*_1_, *l*_2_ and *l*_3_, keeping other parameters constant, as shown in Table 1. The large SECARS EF with the order of ~10^12^ can be achieved based on multi-plasmon resonance.

**Table 1:** Evaluation of the SECARS EF with the different configuration parameters.

Structure	*l*_1_(nm)	*l*_2_(nm)	*l*_3_(nm)	Anti-Stokes		Pump		Stokes		G_SECARS_
				*λ*(nm)	*g*_*as*_	*λ*(nm)	*g*_*p*_	*λ*(nm)	*g*_*s*_	
1	125	270	200	718	11.2	800	29.3	885	60.7	3.4*10^11^
2	100	240	200	730	21.5	821	37	1010	56.8	2.8*10^12^
3	114	294	200	714	16.6	800	52.9	912	59.7	7.9*10^12^

**Figure 8:**
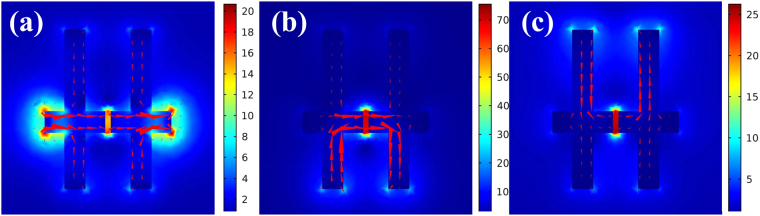
The simulated distributions of the electric field intensity and current distribution at the three different resonance wavelengths: (**a**) *λ*_1_ = 654 nm, (**b**) *λ*_2_ = 846 nm, (**c**) *λ*_3_ = 1048 nm, corresponds to anti-Stokes, pump and Stokes wavelength, respectively.

Also, we have calculated magnitude of the SECARS EF for a Raman mode of molecules at 2278 cm^−1^. We tune the geometric parameters of nanostructure so that middle resonance peak corresponds to the frequency of the pump laser, then the dipole resonance *mode 1* and another resonance peak correspond to the anti-Stokes and Stokes regions in the spectra, respectively. When *l*_1_ = 83 nm, *l*_2_ = 212 nm, *d* = 8 nm and *m* = 34 nm, we can choose 848 nm pump laser, and the anti-Stokes and Stokes wavelengths are 654 nm and 1048 nm, respectively. The electric field distribution at three different wavelengths is shown in the Fig. 8.

It clearly shows that the electric field can be localized at the same gaps at three different resonance wavelengths. Based on formula (5), we can calculate the corresponding CARS signal EF about ~10^13^ ($${G}_{SECARS}={75.1}^{4}\times $$$${21.2}^{2}\times {26.5}^{2}\approx 1.0\times {10}^{13}$$). This result suggests that crisscross dimer nanostructure have great promise to achieve huge electromagnetic field enhancement for single-molecule detection.

## Conclusions

In this paper, we have studied and analyzed the crisscross dimer in the scattering system. The crisscross dimer exhibit Fano resonances via the destructive interference between an electric dipole mode and magnetic dipole modes supported by different subelements. Its scattering character is strongly depending on the length of the *l*_2_ and *l*_3_. Also, it has been proved that the LC circuit model can be used to predict the magnetic dipole resonances in this structure, which plays an important role for the design and adjustment of structure, and provides a new method for structural design in the future scientific research process. We have shown that the multi-resonance electric “field hotpots” can be localized at the same space position, which can be use to achieve a huge CARS signal field enhancement, up to 10^13^. Thence, the crisscross dimer have an important guiding significance for detectors or other optical devices.
