# Author Correction: An engineered CARS substrate with giant field enhancement in crisscross dimer nanostructure

**DOI:** 10.1038/s41598-019-55878-1

**Published:** 2019-12-12

**Authors:** Jia Zhang, Shu Chen, Junqiao Wang, Kaijun Mu, Chunzhen Fan, Erjun Liang, Pei Ding

**Affiliations:** 1 School of Physical Science and Engineering and Key Laboratory of Materials Physics of Ministry of Education of China, Zhengzhou University, Zhengzhou, 450001 China; 2 Department of Mathematics and Physics, Zhengzhou Institute of Aeronautical Industry Management, Zhengzhou, 450015 China

Correction to: *Scientific Report* 10.1038/s41598-017-18821-w, published online 15 January 2018

This Article contains errors in Figure 6: the axes are incorrectly labelled. The correct Figure 6 appears below as Figure 1. As a result, the Figure Legend,

**Figure 1:**
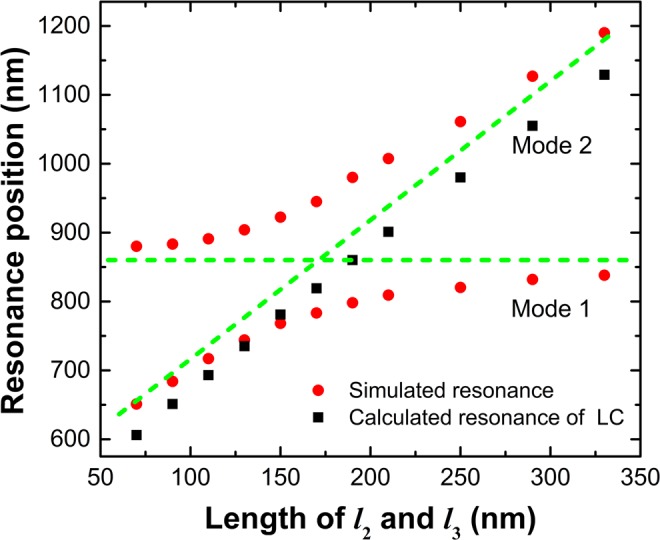
Dependence of resonance wavelength on the length of *l*_*2*_ and *l*_*3*_ (*l*_*2*_ = *l*_*3*_). The red dots indicate the simulated resonance positions of *mode* *1* and *mode* *2*, and the black rectangles represent the calculated resonance positions of *mode* *2* based on LC equivalent circuit model. When the *l*_*2*_ changes, the *l*_*3*_ varies simultaneously with *l*_*2*_, and they always remain the same length (i.e. *l*_*2*_ = *l*_*3*_).

“Dependence of resonance wavelength on the length *l*_*2*_ and *l*_*3*_. The red dots indicate the simulated resonant *mode 1* and *mode 2*, the black rectangles represent the calculated resonance.”

should read:

“Dependence of resonance wavelength on the length of *l*_*2*_ and *l*_*3*_ (*l*_*2*_ = *l*_*3*_). The red dots indicate the simulated resonance positions of *mode 1* and *mode 2*, and the black rectangles represent the calculated resonance positions of *mode 2* based on LC equivalent circuit model. When the *l*_*2*_ changes, the *l*_*3*_ varies simultaneously with *l*_*2*_, and they always remain the same length (i.e. *l*_*2*_ = *l*_*3*_).”

